# Shotgun Cloning of Transposon Insertions in the Genome of *Caenorhabditis elegans*

**DOI:** 10.1002/cfg.392

**Published:** 2004-04

**Authors:** Alexander M. van der Linden, Ronald H. A. Plasterk

**Affiliations:** Hubrecht Laboratory Uppsalalaan 8 Utrecht 3584 CT The Netherlands

## Abstract

We present a strategy to identify and map large numbers of transposon insertions in the genome of *Caenorhabditis elegans*. Our approach makes use of the mutator strain *mut-7*, which has germline-transposition activity of the Tc1/mariner family of transposons, a display protocol to detect new transposon insertions, and the availability of the genomic sequence of *C. elegans*. From a pilot insertional mutagenesis screen, we have obtained 351 new Tc1 transposons inserted in or near 219 predicted *C. elegans* genes. The strategy presented provides an approach to isolate insertions of natural transposable elements in many *C. elegans* genes and to create a large-scale collection of *C. elegans* mutants.


**Comparative and Functional Genomics**
*Comp Funct Genom* 2004; **5**: 225–229.
Published online in Wiley InterScience (www.interscience.wiley.com). **DOI:** 10.1002/cfg.392

**Short Communication**

### Shotgun cloning of transposon insertions in the genome of *Caenorhabditis elegans*

Alexander M. van der Linden and Ronald H. A. Plasterk*
*Hubrecht Laboratory, Uppsalalaan 8, 3584 CT, Utrecht The Netherlands*

*Correspondence to: Ronald H. A. Plasterk, Hubrecht Laboratory, Uppsalalaan 8, 3584 CT, Utrecht, The Netherlands. E-mail: plasterk@niob.knaw.nl*

Received: 15 April 2003
Revised: 21 January 2004
Accepted: 28 January 2004

### Abstract

**We present a strategy to identify and map large numbers of transposon insertions in the genome of *Caenorhabditis elegans*. Our approach makes use of the mutator strain *mut-7*, which has germline-transposition activity of the Tc1/mariner family of transposons, a display protocol to detect new transposon insertions, and the availability of the genomic sequence of *C. elegans*. From a pilot insertional mutagenesis screen, we have obtained 351 new Tc1 transposons inserted in or near 219 predicted *C. elegans* genes. The strategy presented provides an approach to isolate insertions of natural transposable elements in many *C. elegans* genes and to create a large-scale collection of *C. elegans* mutants. Copyright © 2004 John Wiley & Sons, Ltd.**

**Keywords:** ***Caenorhabditis elegans*; insertional mutagenesis screen; *mut-7*; Tc1; transposon insertion display protocol**

### Introduction

The genomic sequence of the genetic model organism *Caenorhabditis elegans* is essentially complete and more than 19 000 genes are predicted (Consortium, 1998). To date, mutants that disrupt gene function have been obtained for less than 10% of these genes. Genome-wide RNAi screens have analysed approximately 86% of the predicted genes in the *C. elegans* genome, and in total 10.3% loss-of-function RNAi phenotypes have been obtained (Kamath *et al.*, 2003). However, although studying gene function by RNAi is advantageous, in many cases a real genetic knock-out is essential. One approach for generating large numbers of mutations in *C. elegans* genes is to create mutations randomly throughout the genome using a transposon-based insertional mutagenesis screen; a method that has proved to be successful in yeast, flies and mice (Zambrowicz *et al.*, 1998; Liao *et al.*, 2000; Vidan and Snyder 2001).

The natural transposable elements of *C. elegans* are effective tools to inactivate genes; they can also be used as genetic markers to positionally clone mutations (Williams *et al.*, 1992; Korswagen *et al.*, 1996). In the genomic sequence of the commonly used Bristol N2 *C. elegans* strain, multiple copies of the Tc1/mariner transposon family are present, e.g. there are at least 31 copies of Tc1, 22 copies of Tc3, and four copies of Tc5 (Fischer *et al.*, 2003). The copy number of the Tc elements is stable, because transposition is inactive in the germline of the Bristol N2 strain. Mutator strains, such as *mut-7* (Ketting *et al.*, 1999), show active germline transposition and have been effectively used in both forward and reverse genetic approaches to inactivate genes with transposon insertions (Plasterk and Luenen, 1997). Transposon insertions do not always result in gene inactivation, due to the fact that they are inserted in introns, but sometimes insertions in exons are also ineffective because they are spliced out of the mRNA (Rushforth and Anderson, 1996). If this is the case, the insertion can be used to generate a deletion derivative that results from imprecise excision (Zwaal *et al.*, 1993). Previously, two approaches have been described to identify a large number of transposon insertions and to map these insertions to the genomic sequence of *C. elegans*. In the first approach, transposon insertions present in mutator strains with a high Tc1 copy number

Copyright © 2004 John Wiley & Sons, Ltd.

were identified by shotgun sequencing (Korswagen *et al.*, 1996). The other approach identified new transposon insertions in a *mut-7* mutator background by a transposon display protocol (Wicks *et al.*, 2000). This method has been modified to circumvent the use of radioactivity and polyacrylamide gel steps (Martin *et al.*, 2002).

In this study, we applied the original transposon display protocol (Wicks *et al.*, 2000) to identify new Tc1 insertions in a limited set of worm lines carrying the *mut-7* mutation that were maintained over 30 generations and to map these insertions to the genomic sequence of *C. elegans*. From a pilot mutagenesis screen, we have obtained 351 new Tc1 insertions that are located in or near 219 predicted genes of *C. elegans*. We show that this approach can be used to generate a large collection of *C. elegans* mutants.

### Materials and methods

### Nematode strains, culturing and manipulation

General methods used for culturing, manipulation and genetics of *C. elegans* were as described (Lewis and Fleming, 1995). All experiments were performed at 20 °C. The following strains were used in this study: Bristol N2 and NL917 [*mut-7(pk204)*III] (Ketting *et al.*, 1999). *mut-7(pk204)* was four times outcrossed using Bristol N2. After the outcrosses, 20 single hermaphrodites were selected, placed on separate *Escherichia coli* OP50-seeded NGM agar plates and grown for two generations. Independent worm strains were collected in M9 buffer (6 g/l $Na_2HPO_4$, 3 g/l $KH_2PO_4$, 5 g/l NaCl, 0.25 g/l $MgSO_4 \cdot 7H_2O$) and frozen at −80 °C using freezing medium (0.1 M NaCl, 0.05 M $KH_2PO_4$, 33% glycerol). These were called NL3100–NL3119. The strain NL3115 was chosen for the pilot mutagenesis screen and homozygosity of the *mut-7* allele was confirmed by sequencing amplified genomic DNA.

### Maintenance of worm lines

In total, 110 single *mut-7* hermaphrodites (NL3115) were picked on 9 cm *E. coli* OP50-seeded NGM-agar plates to start parallel lines that were maintained by transferring a very small piece of agar (containing several hermaphrodites) every 5–6 days to new 9 cm plates (each transfer is about two generations). After 30 generations, parallel worm lines were started with a single hermaphrodite and grown for an additional two generations. Half of each plate was used to freeze worms at −80 °C, while the other half of the plate was used for genomic DNA isolation, as described (Zwaal *et al.*, 1993). The DNA concentration was estimated on a 1% agarose gel.

### Detection of the transposon insertions

The method for visualizing and detecting transposon insertions is as described (Wicks *et al.*, 2000) and is also available on the internet (**http://www.niob.knaw.nl/researchpages/plasterk/transdip.html**). In short, extracted genomic DNA (100 ng) was digested with the enzyme *Sau3* A (10 U; New England Biolabs) for several hours at 37 °C. The enzyme was heat-inactivated (20 min at 65 °C) and an oligonucleotide-vectorette cassette (503: 5′-GATCCAAGGAGAGGACGCTGTCT-GTCGAAGGTAAGGAACGGACGAGAGAAG-GGAGA; 504: 5′-TCTCCCTTCTCGAATCGTAA-CCGTTCGTATACGAGAATCGCTGTCCTCTC-CTTG; 15 pmol) was ligated (overnight at 16 °C) to the digested genomic DNA in a 100 μl total reaction volume, using T4 DNA ligase (10 U, Boehringer Mannheim). An aliquot of ligated DNA (3 μl) was used in the first round of PCR using primers for the Tc1 terminus (Tc1L1: 5′-TGTTCGAAGCCAGCTTACAATGGC or Tc1R1: 5′-GCTGATCGACTCGAGCCACGTCG; 10 pmol) and a primer for the vectorette (505: 5′-CGAATCG-TAACCGTTCGTACGAGAATCGCT; 10 pmol). Products of the first round of PCR were 100× diluted, and 2 μl was used for the second round of PCR, using nested primers for the vectorette (337NEW: 5′-GTACGAGAATCGCTGTCCTC; 10 pmol) and the Tc1 terminus. The Tc1 primer is radiolabelled with $^{32}P$ $\gamma$ATP (Tc1L2: 5′-TCAAGT-CAAATGGATGCTTGAG or Tc1R2: 5′-GATTTT-GTGAACACTGTGGTGAAG; 1 pmol). In all PCR steps, 25 cycles were performed with 1 min at 95 °C, 1 min at 58 °C and 1 min at 72 °C. The final volume of the PCR reaction was 25 μl using *Taq* DNA polymerase (1 U; Gibco BRL). The products from the latter PCR reaction were separated on a 6% denaturing polyacrylamide gel (Accugel™ 19 : 1; National Diagnostics) with 0.25 M NaAc in the lower buffer compartment to reduce the

Copyright © 2004 John Wiley & Sons, Ltd. 

spacing between the bands of the gel. We used 0.35 mm mylar spacers and combs (S2 model, Life Technologies) for the electrophoresis (1.5–5 h at 65 W). The gel was dried on GB002 blotting paper (Schleicher and Scheull) with fluorescent markers for orientation and exposed to a Super RX film (Fuji). Every lane on the autodiagram shows a pattern of bands corresponding to transposon insertions present in the *C. elegans* genomic sequence of a single worm line. Each new band was cut from the polyacrylamide display gel, put into 25–50 µl water and heated for 10 min at 65 °C. An aliquot of 1–2 µl was used for a PCR with nested primers for the vectorette (337NEW; 10 pmol) and for the Tc1 terminus (Tc1L2 or Tc1R2; 10 pmol) using the same PCR conditions. PCR products were purified with a Whatman 384-well DNA-binding filter plate. Sequence reactions were done using a ABI PRISM Big Dye terminator sequencing kit, using the Tc1R2 primer, and were analysed on a ABI 3700 DNA analyser.

### Results and discussion

We made use of the mutator strain *mut-7* for the pilot insertional-mutagenesis screen, since *mut-7* activates multiple transposons in the germline, including Tc1, Tc3 and Tc5 (Ketting *et al.*, 1999). One technical difficulty for the screen is that the *mut-7* strain we used has at least 60, if not more, interspersed copies of Tc1 that have arisen by insertions into new genomic sites. To reduce this number, we first outcrossed *mut-7*(*pk204*) four times with Bristol N2 and subsequently froze 20 independent *mut-7* strains (NL3100–NL3119). To determine which strain has the lowest transposon copy number, we used the transposon display technique to visualize new transposon insertions (Wicks *et al.*, 2000). We found that the *mut-7* outcrossed strain NL3115 has the lowest Tc1 copy number ($35 \pm 5$).

To generate a large set of new Tc1 insertions, we started 110 parallel lines of the *mut-7* strain NL3115 by cloning the progeny of a few *mut-7* hermaphrodites. These lines were maintained over 30 generations, and subsequently one hermaphrodite/line was picked and grown for an additional two generations. In total, 86 of the 110 parallel worm lines were frozen to be included in the pilot mutant collection, and an aliquot was used to extract genomic DNA. To determine how many Tc1 insertions arise over generations, we also extracted genomic DNA from 14 of the 110 parallel lines of the *mut-7* strain NL3115 after 8, 18 and 30 generations. We counted the number of new Tc1 insertions by amplifying either the right- or the left-flanking sequence of Tc1, which were visualized by the transposon display technique using a defined region of the gel. As shown in Table 1, we found a linear increase of new Tc1 insertions that arose after 8, 18 and 30 generations. On average, 12 new Tc1 insertions/line were observed after maintaining these lines for at least 30 generations (Table 1). Based on the average number of Tc1 insertions after 30 generations, more than 1000 new Tc1 insertions are expected in the 86 parallel worm lines.

From the 86 parallel worm lines of the pilot mutant collection, we visualized the right-flanking sequences of Tc1 in each line with the transposon insertion display technique (Wicks *et al.*, 2000). To determine the genomic insertion site, each new band corresponding to a Tc1 insertion was excised from the gel, reamplified by PCR and sequenced. Subsequently, the flanking sequences were compared to the genomic sequence of *C. elegans* using the BLAST program (**http://www.sanger.ac.uk/Projects/C_elegans/blast_server.shtml**). To date, we have obtained 351 sequences flanking the Tc1 transposon. A list of the isolated Tc1 insertions is given in Supplemental Table 1 at: **http://www3.interscience.wiley.com/cgi-bin/jabout/77002016/OtherResources.html** and is also submitted for integration in Wormbase: **http://www.wormbase.org**

Although exonic DNA regions accounts for 27% of the genome (Consortium, 1998), we only found 12% of the Tc1 insertions in predicted exons; 28% of the insertions are found in intronic regions and 19% in 5′- or 3′-untranslated regions as defined by 500 bp upstream or downstream of the coding sequence based on Wormbase annotation. The remaining Tc1 insertions are located in intergenic regions. A probable explanation for the strong selection against Tc1 insertions within coding regions is that coding sequences are higher in GC content (Waterston *et al.*, 1997) because the canonical target sequence of Tc1 is TA. We also found that the Tc1 insertions are distributed all over the genome of *C. elegans* and no bias for any genomic region is observed (Supplemental

Copyright © 2004 John Wiley & Sons, Ltd.

**Table 1.** New Tc1 insertions that arise over a number of generations

| | 8th n | | 18th n | | 30th n | |
|---|---|---|---|---|---|---|
| **Worm line** | **Left Tc1** | **Right Tc1** | **Left Tc1** | **Right Tc1** | **Left Tc1** | **Right Tc1** |
| 1 | 4 | 3 | n.d. | n.d. | n.d. | n.d. |
| 2 | n.d. | n.d. | 5 | 7 | 8 | 8 |
| 3 | 9 | 7 | 7 | 8 | 12 | 15 |
| 4 | 3 | 3 | 3 | 4 | 7 | 9 |
| 5 | 5 | 2 | 10 | 3 | n.d. | n.d. |
| 6 | n.d. | n.d. | 9 | 11 | n.d. | n.d. |
| 7 | 3 | 1 | n.d. | n.d. | n.d. | n.d. |
| 8 | 4 | 3 | n.d. | n.d. | 13 | 8 |
| 9 | 2 | 4 | 3 | 5 | n.d. | n.d. |
| 10 | n.d. | n.d. | n.d. | n.d. | 6 | 9 |
| 11 | 4 | 6 | n.d. | n.d. | 14 | 17 |
| 12 | 2 | 4 | n.d. | n.d. | n.d. | n.d. |
| 13 | 1 | 1 | 4 | 4 | 12 | 17 |
| 14 | n.d. | n.d. | 6 | 5 | 16 | 18 |
| AVG | 3.7 | 3.4 | 5.9 | 6 | 11 | 12.6 |

In total, 14 parallel worm lines were maintained for 8, 18 and 30 generations (n), and an aliquot of each worm line was used to extract genomic DNA (see Material and methods). The right- and left-flanking DNA sequence of the Tc1 transposon was visualized by the transposon insertion display protocol, as described (Wicks *et al.*, 2000). The number of new Tc1 insertions observed in a defined region of the gel was counted. The average (AVG) of new Tc1 insertions of the 14 worm lines is given. n.d., not determined.

Table 1). In addition to Tc1, there are multiple copies of Tc3 and Tc5 present in the *C. elegans* genome, and *mut-7* also activates the transposition of these copies (Ketting *et al.*, 1999). Thus, also new Tc3 and Tc5 insertions in the 86 parallel lines could be isolated; we have not yet analysed their abundance in these lines.

To assay whether we could recover any insertions in genes, we thawed and singled out worms that contain a predicted insertion in the gene *sir-2.1* (R11A8.4) and *gpa-5* (F53B1.7). Each single worm was tested by PCR with primers corresponding to the Tc1 terminus and primers in the gene containing the Tc1 insertion. In this manner, we could recover homozygous animals of *sir-2.1* and *gpa-5* that carry a Tc1 insertion. No phenotype is expected for *gpa-5* because the Tc1 insertion is located in the 6th intron of *gpa-5*, but the Tc1 insertion located in the 5th exon of *sir-2.1* does disrupt protein expression and confers phenotypic effects (Viswanathan and Guarente, unpublished results).

Recently, another transposon-based pilot insertional mutagenesis screen was described that used a modification of the transposon insertion display protocol to prevent the use of radioactive and polyacrylamide gels, and instead visualized new insertions on an agarose gel (Martin *et al.*, 2002). However, in comparison to the polyacrylamide gels (6%) we used, agarose gels (2%) are lower in resolution and may not detect all insertions. For instance, Martin and co-workers generated 862 worm lines using a non-outcrossed *mut-7* strain with, on average, two detectable insertions/line that were maintained for 10 generations. In contrast, we generated 86 worm lines that carry, on average, about four detectable insertions/line after 10 generations (Table 1). For downstream handling (e.g. deposition in strain collections) it may be advantageous to use a method that detects the highest number of transposons/strain, and this may outweigh the relative practical disadvantage of the use of a radioactive label during the screening phase.

In conclusion, this pilot mutagenesis screen was done to analyse the feasibility of creating a large-scale collection of genetic *C. elegans* mutants. In this pilot study, we found 351 new Tc1 insertions in or near 219 predicted *C. elegans* genes. These results suggest that when starting from 1000 parallel worm lines and maintaining these lines for over 30 generations, 12 000 new Tc1 insertions can be expected. More than one-third of these insertions will be in coding and intronic sequences, enough to hit about 20% of the predicted *C. elegans* genes, of which only about 30% will affect gene function

Copyright © 2004 John Wiley & Sons, Ltd. 

with respect to insertions in coding regions. The non-directed transposon-based mutagenesis screen presented in this study can provide an approach to knock out many genes of *C. elegans* as an alternative or an addition to gene-directed approaches.

### Acknowledgements

This work was supported by the Netherlands Organization for Scientific Research (NWO), grant 014-80-008. We thank Edwin Cuppen and Hendrik C. Korswagen for critical reading of the manuscript.

### References

*C. elegans* BLAST server: **http://www.sanger.ac.uk/Projects/C_elegans/blast_server.shtml**

Consortium. 1998. Genome sequence of the nematode *C. elegans*: a platform for investigating biology. The *C. elegans* Sequencing Consortium. *Science* **282**: 2012–2018.

Fischer SEJ, Wienholds E, Plasterk RHA. 2003. Continuous exchange of sequence information between dispersed Tc1 transposons in the *C. elegans* genome. *Genetics* **164**: 127–134.

Kamath RS, Fraser AG, Dong Y, *et al*., 2003. Systematic functional analysis of the *Caenorhabditis elegans* genome using RNAi. *Nature* **421**: 231–237.

Ketting RF, Haverkamp TH, van Luenen HG, Plasterk RH. 1999. Mut-7 of *C. elegans*, required for transposon silencing and RNA interference, is a homolog of Werner syndrome helicase and RNaseD. *Cell* **99**: 133–141.

Korswagen HC, Durbin RM, Smits MT, Plasterk RHA. 1996. Transposon Tc1-derived, sequence-tagged sites in *Caenorhabditis elegans* as markers for gene mapping. *Proc Natl Acad Sci USA* **93**: 14 680–14 685.

Lewis JA, Fleming JT. 1995. Basic culture methods. *Methods Cell Biol* **48**: 3–29.

Liao GC, Rehm EJ, Rubin GM. 2000. Insertion site preferences of the P transposable element in *Drosophila melanogaster*. *Proc Natl Acad Sci USA* **97**: 3347–3351.

Martin E, Laloux H, Couette G, *et al*., 2002. Identification of 1088 new transposon insertions of *Caenorhabditis elegans*: a pilot study toward large-scale screens. *Genetics* **162**: 521–524.

Plasterk RHA, van Luenen H. 1997. Transposons. In *C. elegans II,* Riddle D, Blumenthal T, Meyer B, Priess J (eds). Cold Spring Harbor Laboratory Press: New York; 97–116.

Rushforth AM, Anderson P. 1996. Splicing removes the *Caenorhabditis elegans* transposon Tc1 from most mutant pre-mRNAs. *Mol Cell Biol* **16**: 422–429.

Transposon Insertion Display Protocol: **http://www.niob.knaw.nl.researchpages/plasterk/transdip.html**

Vidan S, Snyder M. 2001. Large-scale mutagenesis: yeast genetics in the genome era. *Curr Opin Biotechnol* **12**: 28–34.

Waterston RH, Sulston JE, Coulson AR. 1997. The genome. In *C. elegans II*, Riddle D, Blumenthal T, Meyer B, Priess J (eds). Cold Spring Harbor Laboratory Press: New York; 23–45.

Wicks SR, de Vries CJ, van Luenen HG, Plasterk RH. 2000. CHE-3, a cytosolic dynein heavy chain, is required for sensory cilia structure and function in *Caenorhabditis elegans*. *Dev Biol* **221**: 295–307.

Williams BD, Schrank B, Huynh C, Shownkeen R, Waterston RH. 1992. A genetic mapping system in *Caenorhabditis elegans* based on polymorphic sequence-tagged sites. *Genetics* **131**: 609–624.

Wormbase Database: **http://www.wormbase.org**

Zambrowicz BP, Friedrich GA, Buxton EC, *et al*. 1998. Disruption and sequence identification of 2000 genes in mouse embryonic stem cells. *Nature* **392**: 608–611.

Zwaal RR, Broeks A, van Meurs J, Groenen JT, Plasterk RH. 1993. Target-selected gene inactivation in *Caenorhabditis elegans* by using a frozen transposon insertion mutant bank. *Proc Natl Acad Sci USA* **90**: 7431–7435.

Copyright © 2004 John Wiley & Sons, Ltd. 
